# Developing a theory-based behavior change intervention to improve the prescription of surgical prophylaxis

**DOI:** 10.1007/s11096-021-01338-8

**Published:** 2021-11-20

**Authors:** Anna Leena Lohiniva, Iman Heweidy, Samiha Girgis, Omar Abouelata, Caroline Ackley, Shady Samir, Maha Talaat

**Affiliations:** 1 WHO Eastern Mediterranean Regional Office, Cairo, Egypt; 2 Ain Shams University Hospitals, Cairo, Egypt; 3 WHO Egypt Country Office, Cairo, Egypt; 4 London School of Tropical Medicine and Hygiene, London, England, UK; 5 Brighton and Sussex Medical School, Brighton, England, UK

**Keywords:** Behavior change intervention, Behavior change wheel, Surgical prophylaxis, Theoretical Domains Framework

## Abstract

*Background* Antimicrobial resistance (AMR) is increasingly pervasive due to multiple, complex prescribing and consuming behaviours. Accordingly, behaviour change is an important component of response to AMR. Little is known about the best approaches to change antibiotic use practices and behaviours. *Aim* This project aims to develop a context-specific behaviour change strategy focusing on promoting appropriate prescription practices following the World Health Organization recommendations for surgical prophylaxis in an orthopaedic surgery unit in Egypt. *Method* The project included a formative qualitative research study with 31 in-depth interviews with orthopaedic surgeons that was based on the Theoretical Domains Framework (TDF) and an intervention that was developed to following the Behaviour Change Wheel (BCW) in a knowledge co-production workshop with ten public health experts that ensured that the theory based intervention was a culturally acceptable, practical and implementable intervention. *Results* The prescription of surgical prophylaxis was influenced by eight TDF domains from which workshop participants selected five to be included in the behaviour change intervention including, knowledge, belief in consequences (mistrust towards infection prevention and control measures), environmental factors (lack of prescription guidelines), professional role and reinforcement (a lack of appropriate follow up actions influenced prescription of surgical prophylaxis). The appropriate set of behaviour change functions of BCW and related activities to improve the current practices included education, enablement, persuasion, environmental restructuring and restriction. *Conclusion* The study showed that a theory based, and context specific intervention can be created by using the TDF and BCW together with knowledge-co creation to improve the prescription of surgical prophylaxis in and Egyptian orthopaedic unit. The intervention needs to piloted and scaled up.

## Impacts on practice

- A systematic and replicable approach to identifying the determinants of the prescription of surgical prophylaxis in a low resource setting has been developed.
- Framing of the determinants, in terms of the Theoretical Domains Framework (TDF) and identification of the behavior change intervention using the Behaviour Change Wheel (BCW) approach, allow specific recommendations to be made for intervention design and future research in this area.
- Knowledge co-creation helps to assess the feasibility and acceptability of the results in the given context.

## Introduction

The misuse and overuse of antibiotics are major driving forces of AMR worldwide [1, 2]. New AMR mechanisms are emerging and spreading globally, threatening our ability to treat common infectious diseases, resulting in prolonged illness, disability, and death. The COVID-19 pandemic has increased the use of antibiotics in the treatment of infected patients and is likely to increase AMR further worldwide [3]. About 50% of all antimicrobials prescribed globally are unnecessary [4], where antibiotic use has increased by 65% between 2000 and 2015, which is a key concern for public health [5]. The WHO AMR Global Action Plan calls for countries to improve the awareness and understanding of AMR through effective communication, education, and training. Changing behaviours of prescribers is an important piece of this puzzle. Many social and cultural factors influence antibiotic prescribing practices in acute care settings. These need to be understood in their given context in order to promote behaviour change [6, 7]. Behaviour change interventions that only rely on education or policy change often have limited impact on changing behaviours [8], and should be supplemented by addressing the cultural factors influencing prescribing practices.

The WHO Eastern Mediterranean regional office (EMRO) conducted a series of behavior change pilots in Egypt, Jordan [9] and Sudan between 2017 and 2019 to advance in the behaviour change programming to reduce the spread of antibiotic resistance in the region. Accordingly a theory-based behaviour change model called “Tailoring Antimicrobial Resistance Programme (TAP)” was adapted from earlier behaviour change guides (TIP-TAP EURO) [10]. The model focused on addressing the underlying contextual factors of behaviours related to a specific problem of antibiotic prescription identified by each country. These pilots aimed to assist WHO to implement behaviour change intervention locally and to frame generic approaches that feed into regional guidance on to use context specific and behavioural insights-oriented methods to improve antibiotic prescribing related behaviours. The TAP methodology draws on the Theoretical Domains Framework (TDF) to identify behaviour change domains and uses the Behaviour Change Wheel (BCW) as a theory of change. TDF has proven to be a practical framework for public health professionals who are not necessarily experts in complex psychological behaviour change theories to develop an in-depth understanding of behaviours [11]. BCW represents an ideal match to TDF as the latter’s results can be merged with the BCW behaviour change functions [8]. Inappropriate antibiotic prescribing before and after surgery is widely reported worldwide, including in Egypt where prolonged post discharge treatment in particular has shown to be a major problem [12–17].

### Aim

This paper describes the TAP methodology development process in Egypt that was initiated in 2018 as a collaborative activity between the Ain Shams University Hospitals (ASUH) and the WHO. The study aimed to modify surgical prophylaxis prescription practices in the orthopaedic surgery department of the ASUH in alignment with the WHO Guidance for surgical prophylaxis [18].

## Ethics approval

The project and TAP methodology received ethical approval by the Ain Shams University ethical review board in February 2, 2018, followed by the approval of the WHO Eastern Mediterranean Regional Office Ethical Board March 15, 2018. In addition, on the 17 of May in 2019 the London School of Tropical Medicine and Hygiene (LSTMH) MSc Research Ethics Committee approved the protocol for MSc in Public Health program with a reference number 16327. All participants signed consent to participate.

## Method

This project is based on two distinct methods that comprise the TAP methodology: (1) qualitative research to explore the factors influencing the prescription for surgical prophylaxis among orthopaedic surgeons at ASUH; and (2) a workshop with select ASUH staff to develop a context specific behaviour change intervention. The data was collected in June and July 2019 and the workshop were conducted in October 2019.

The study used the theoretical domains framework to explore the underlying causes of the inappropriate antibiotic prescription for surgical prophylaxis amongst orthopedic surgeons at ASUH. TDF consists of 14 domains: knowledge, skills, social environment, professional role, memory and decision-making process, environmental factors, goals, intentions to change, optimism, reinforcement, behaviour regulation, beliefs in capabilities, beliefs in consequences, and emotions [11]. The domains were defined for the topic and context of the study. A semi-structured question guide was developed to explore which domains were relevant for the prescription of antibiotics for surgery and how they influence behaviour.

A field team of a cultural anthropologist and two IPC professionals trained in qualitative data collection conducted 35 in-depth interviews (IDI) during a period of 2 months in 2018. IDIs were conducted with orthopedic surgeons at ASUH. The team members conducted a maximum of two interviews per day and each interview lasted for 25–40 min. The study sample included one female and 30 male orthopaedic surgeons who were staff members in the ASUH. Half of the surgeons were residents whose working experience ranged from 1 year to 3 years and the other half were consultants whose work experience ranged from 10 to 22 years. A purposive sample was selected to gain diversity by including senior and junior surgeons representing different orthopedic subspecialties. Data saturation was the principle for obtaining an adequate sample size [19].

Informed verbal consent was obtained from participants. Interviews were recorded and conducted in a private space in the hospital selected by the participant, and no identifiers were obtained to ensure confidentiality. Audio recordings of the interviews were transcribed verbatim and translated from Arabic to English by the team members. Debriefing sessions were conducted weekly for the team to share key findings and to discuss modification of interview techniques and questions as necessary.

The first author of this paper (AL) conducted thematic data analysis according to the TDF domains [11]. Thematic analysis included familiarization of the data by reading the transcripts several times to gain an initial understanding of the data followed by manual coding that lead to the development of a code book that was shared by other field team members to get a consensus of the final set of codes. AL developed a chart where the codes were organized based on TDF domains and continued the analysis process by identifying categories and patterned meanings across the dataset. The final interpretation of the data included a set of overarching themes used as a base for developing a behaviour change intervention [20].

A 3-day knowledge co-production workshop was held with 10 public health experts from ASUH and the WHO to design an intervention that is acceptable, practical and implementable utilizing the findings from both the qualitative study and literature on global best practices to improve antibiotic prescription practices [21, 22].

The workshop used the Behaviour Change Wheel as a framework to develop the behaviour change intervention. The BCW is based on three domains which interact to explain behaviour: capability, opportunity, and motivation. They further link with the domains of TDF. Capability includes the TDF domains of knowledge, skills, memory and habits, and behavioural regulations. Opportunity incudes the TDF domains of social and environmental factors, whereas motivation will include the remaining TDF domains. Each BCW domain is matched with a set of intervention functions and policy that are expected to be effective for bringing about desired changes in behaviour.

The workshop resulted in development of a behaviour change strategy including selected behaviour domains and linked intervention functions and activities to be piloted. This strategy aimed to promote prescription practices for surgical prophylaxis in the orthopaedic surgery department at ASUH.

## Results

In this section we describe the findings of (1) the qualitative research study and (2) the multi-disciplinary knowledge co-production workshop.

### Qualitative study results

The factors identified during the interviews that influence the prescription of surgical prophylaxis were linked to eight out of fourteen domains of the TDF framework, including knowledge, beliefs in consequences, communication skills, professional role, psychological reactions, environmental context, optimism, and reinforcement.

### Domain: knowledge

Respondents discussed how their limited knowledge influenced the prescription of antibiotics for surgical prophylaxis. *“This is what I learned during my residency by observing others and my superiors. This is what we all do”- Orthopaedic surgeon, resident*.

### Domain: belief in consequences

Respondents discussed AMR as a consequence of improper prescription of antibiotics for surgical prophylaxis. However, they had given little thought to their own antibiotic prescription practices as a contributing factor to AMR. Instead, they blamed community pharmacists and patients for the overuse. For example, one Orthopaedic surgeon, consultant said *“All I can say is that this is a big problem in Egypt. People and pharmacists use antibiotics without any control whatsoever.”*

Many respondents were aware about the international guidelines for antibiotic prescription; however, they did not believe that these guidelines were applicable in the Egyptian context because they believed that international guidelines were developed in the context of western countries where AMR was less of a problem than in Egypt.

“In Egypt AMR is a big problem. For that reason, we have to prescribe more stronger antibiotics and for longer periods of time than elsewhere.” (Orthopaedic surgeon consultant)

### Domain: skills

Respondents discussed the c difficulties in answering patient demand for receiving antibiotics following hospital discharge. They acknowledged that the practice was unnecessary nor supported by any evidence, but also thought that halting the prescription of antibiotics after hospital discharge was difficult to be communicated *“Patients tell me what they want. Yes, I discuss with them, but they don’t always listen to me. They may not consider my advice about antibiotics.” (Orthopaedic surgeon consultant).*

### Domain: professional role

Junior doctors highlighted that senior physicians were always the decision makers if they were involved in the prescription for surgical prophylaxis, as indicated by a resident orthopaedic surgeon. *“We follow what senior doctors are recommending.”* It was also highlighted that clinical pharmacists had no role to play in the prescription of antibiotics unless there was a patient with resistant infections*. “We do not benefit from the pharmacological knowledge of clinical pharmacists. We don’t have them.”- Orthopaedic surgeon, consultant.*

However, communication between senior and junior doctors was often limited and, as such, they were left with limited guidance on how to prescribe antibiotics. *“Sometimes we don’t see our consultant at all. He may not follow the case much. I can go back to him if I have a problem” – (Orthopaedic surgeon, resident).*

### Domain: psychological reactions

Respondents discussed the fear of patient acquisition of infection due to the limited confidence in the effectiveness of the hospital’s IPC programme contributed to the practice of antibiotic prescribing. *“Here in the hospital there are no IPC measures implemented. Doctors and nurses practice as they wish. There is no follow up. Where is the IPC team?” (Orthopaedic surgeon, consultant).* Some of the respondents referred to breaches of IPC practices they witnessed, such as entering the operating theatre multiple times with the same personal protective equipment, not using masks or gloves as per guidelines, insufficient sterilization of surgical equipment, breaches in environmental cleaning, and a lack of nursing staff.

The patients were from low socio-economic backgrounds were believed to be prone to infection due to poor general health conditions, nutritional status, and unhygienic living conditions. One resident orthopaedic surgeon explained, *“Our patients are so fragile. It is easy for them to acquire an infection. That’s why we cannot comply with any international guidelines.”*

Many respondents noted that also inadequate hospital infrastructure might be a contributing factor to the acquisition of hospital-acquired infections. Examples are overcrowded wards with lots of visitors who stayed for extended periods of time at bedsides, continuous construction work, and limited air conditioning in different areas of the hospital. Another orthopaedic surgeon, resident noted, *“In a private hospital we have one patient per room, and we have nice furniture and surroundings. But here in the government hospital we have to use antibiotics to kill any germs in the environment.”*

### Domain: environmental context

Respondents also explained that their selection of certain types of antibiotics is influenced by availability. On many occasions, certain types of antibiotics are out of stock and sometimes broad-spectrum antibiotics are also unavailable.

Respondents explained that they did not have guidelines for surgical prophylaxis based on their Egyptian context*, “We don’t follow any guidelines as we don’t have guidelines”- Orthopaedic surgeon, consultant*.

### Domain: optimism

Responses regarding the belief in changes to prescribing practices varied significantly, but most respondents did not believe that change in prescribing behaviour can happen*, “This is our practice. Changes are not possible.”- Orthopaedic surgeon, consultant*. Many explained that senior staff and eminent surgeons in particular will not change their current prescribing practices. Others cited that changes could be introduced when IPC practices were improved*, “Sure, sometime later when our hospital is in better shape and infection control practices are well institutionalized”- Orthopaedic surgeon, consultant.*

### Domain: reinforcement

All respondents agreed that there was no follow up or feedback on their antibiotic prescribing practices including surgical prophylaxis. There is no follow up or peer control*, “*Nobody follows how we prescribe antibiotics. There is no positive or negative feedback*”- Orthopaedic surgeon, consultant.*

### Workshop of knowledge co-production

The workshop resulted in the selection of five behavioural domains to be addressed in the behaviour change intervention based on the qualitative research study, followed by selecting five intervention functions which correspond with the BCW framework and are in line with global best practices. Workshop participants selected the interventions which were evaluated based on their acceptability, practicality and how well they can be implemented on a large-scale basis.

### Domain: knowledge

The qualitative research study indicated that there is lack of knowledge about antibiotic prescription among orthopaedic surgeons. The rapid literature review indicated that educational interventions alone have little impact on behaviour change [23–25]. However, education together with other interventions can have a positive impact on behaviour change [26–28].

The workshop participants discussed educational sessions as culturally acceptable interventions but inviting senior staff to an education session could be interpreted by senior staff as undermining their knowledge and skills. From a practical point of view, organizing educational sessions was considered difficult because doctors were generally busy. Incorporating educational components into existing hospital staff meetings and continuous educational trainings was therefore considered more feasible. The sessions were considered easily implementable even on a larger scale within the hospital. In addition, an educational message in the form of advocacy for AMR was suggested to be incorporated into regular unit meetings where senior staff meets.

### Domain: beliefs in capabilities

The qualitative research study pointed out that one of the major drivers of current misuse of antibiotics was a fear of the acquisition of hospital acquired infections, linked to beliefs about insufficient infection control measures as well as the poor general health and unhygienic living environments of patients. Literature shows that multi-faceted antibiotic stewardship programs including structural adjustments to improve IPC measures showed positive results in changing prescription practices [29, 30].

The BCW intervention concerning reducing the fear of patient acquisition of hospital acquired infections through improvements in IPC corresponds with the TDF theme of ‘enablement,’ which itself refers to modifications in the enabling environment. Accordingly, respondents discussed the need to improve IPC programmes, which is a concrete, visible and measurable action that is likely to be strongly welcomed by all healthcare workers. Workshop participants also believed that enhancing IPC measures would provide an opportunity for the IPC team to become more visible and appreciated. An IPC related intervention was considered practical as ASUH has a large and well-trained IPC staff that can incorporate additional IPC measures to support the orthopaedic surgeons. However, the intervention is likely to require advocacy as IPC related measures are not prioritized by the hospital administration.

### Domain: professional role

The qualitative research study illustrated that the role of senior doctors was critical. The workshop participants highlighted the importance of paying special attention to the senior staff. The BCW intervention function corresponding with professional role was the TDF theme of ‘persuasion,’ and workshop participants agreed to include persuasion by developing appropriate activities such as reminders that address senior staff to emphasize their role in the intervention.

### Domain: environmental factors

The qualitative study revealed that there were no guidelines for a surgical prophylaxis. Although the literature on AMR suggests that introducing antibiotic guidelines and policies alone does not always result in behaviour change, guidelines as a part of an intervention have had a positive impact on improving prescription practices [31–33].

The workshop participants indicated that guidelines can be acceptable if the introduction of them takes place gradually, starting from the senior staff first. Ideally, the senior staff should be involved in the development of the guidelines to ensure they are accepted and applied by all. The workshop participants also agreed that training and orientation on the guidelines is important to ensure implementation. The scaling up of guidelines was also seen as feasible even on a national scale. The workshop participants therefore agreed to include a BCW intervention function “environmental restructuring” that included developing and disseminating hospital-based guidelines for surgical prophylaxis.

### Domain: reinforcement

The qualitative findings showed that there is no follow up of prescription of surgical prophylaxis. The surgeons did not receive any feedback from their peers or the management regarding their antibiotic prescription practices. Yet, the rapid review pointed out that multi-component interventions that includes follow up, audits, or feedback systems were generally effective in changing prescription [34, 35].

The workshop participants discussed how implementing a feedback or audit system would not be easily accepted by the doctors who are used to making decisions about antibiotics independently. They believed that senior staff would be the most difficult, if not impossible, to convince them on the importance of such systems. Therefore, workshop participants suggested that the feedback system include BCW domain ‘restrictions’ that will require surgeons to comply even if they were not in agreement. Restriction systems as part of antibiotic stewardship programs were also used successfully in other international settings [36, 37]. Workshop participants also discussed including a clinical pharmacist in the follow up system as the rapid literature review showed that the involvement of clinical pharmacists is highly beneficial [38–40]. The workshop participants also highlighted that as university hospital in general have similar structures and staffing, the same system can be easily adjusted for other settings.

## Discussion

TAP is an effective method to understand the underlying factors behind antibiotic prescription practices and in developing sound evidence-based behaviour change intervention in the Egyptian context. The TDF used in the qualitative study proved to be a practical framework for collecting qualitative data to understand the prescription practices for surgical prophylaxis in the Egyptian healthcare setting where respondents appreciated the structured way of exploring behaviours.

The qualitative study highlighted that there was a lack of knowledge about proper antibiotic prescription. Number of studies highlights that sufficient knowledge about antibiotics and antimicrobial resistance is a prerequisite for proper prescription practices [41]. However, the qualitative study also identified misconceptions that must be taken into consideration when aiming at improving the prescription of surgical prophylaxis. Firstly, prescribers did not think that their prescription practices were contributing to the AMR problem of the country, which has been found as a misconception also in other countries [41]. Secondly, they believed that international guidelines were not applicable to the Egyptian context where antibiotic consumption was believed to be higher than elsewhere with higher rates of resistant bacteria. It is not uncommon that international guidelines are seen inappropriate in a local context as the definitions of improper antibiotic use differ across various settings and they are often linked with local understanding of uncertainties and moral judgments [42].

The qualitative data also highlighted that mistrust towards IPC programmes were driving fear of infection that made prescribers reluctant to make modifications to their current surgical prophylaxis practices. Breaches in IPC have been commonly identified as a barrier for improving the prescription for surgical prophylaxis in middle- and low-income countries where prescribers tend to prescribe antibiotics for longer periods of time [43]. A study in Egypt indicates that treating physicians are motivated to prescribe antibiotics to avoid potential secondary bacterial infections caused by lack of IPC measures or due to unhygienic living conditions of the patients [44].

Interventions that aim to change practices must fit to the organizational culture to be accepted and sustained [45]. The data also showed that in the Egyptian context, a top bottom approach is appropriate to drive a change; meaning that engaging senior physicians and department directors is essential for any change in the practices of younger physicians. Trust in the perceptions of senior practitioners have been shown to be powerful reasons for the inappropriate prescription of antibiotics in several countries in the world [46, 47].

The knowledge co- production workshop ensured that the selected intervention functions were context appropriate. For example, an intervention was suggested to reduce the fear of physicians for patient acquisition of infection due to limited IPC. This was addressed by enhancing the IPC programme activities to ensure patient safety. This was supplemented by updating the hospital guidance on surgical prophylaxis addressing the needs of the orthopaedic surgeons. Knowledge co-production demonstrated also efficacy in identifying local solutions for activities. For example, as a result of co-production, the intervention activities were merged into existing hospital activities such as incorporating educational sessions on antibiotics and AMR during the regular department meetings that made the intervention low cost. Co-production interventions are known to be valuable in behaviour change programming as they stimulate participation in the design development including co-identification of problems, co-development of alternatives, and co-implementation of solutions that promote mutual learning and co-realization in all stages of the process. To participate in the design process [48, 49].

The study has some limitations that must be acknowledged. The practices reported in interviews might have been influenced by socially desirable answers given the potentially sensitive nature of subjects discussed. As the study is based on qualitative data, there is a need to evaluate critically the applicability of the findings in larger scale implementation.

The future studies include measuring the impact of the intervention and scaling up the model in similar sociocultural settings in the region and elsewhere.

## Conclusion

This study provided important insights into behavioural factors that influence the prescription of surgical prophylaxis in a low resource setting. The incorporation of behavioural theories—TDF and BCW—supported the identification of key factors that are integral to understanding the prescription practices; knowledge, beliefs in capabilities, professional role, environmental context & resources and resources: and intervention functions to modify the current practices: education, enablement, persuasion, environmental restructuring and restriction.
